# ROC1 promotes the malignant progression of bladder cancer by regulating p-IκBα/NF-κB signaling

**DOI:** 10.1186/s13046-021-01935-5

**Published:** 2021-05-07

**Authors:** Qi Wu, Xiaoqing Zhou, Peng Li, Mao Ding, Shengjie You, Zhaoyu Xu, Junjie Ye, Xuedong Chen, Mingyue Tan, Jun Wang, Wei Wang, Jianxin Qiu

**Affiliations:** 1 Department of Urology, The Sixth Affiliated Hospital of Wenzhou Medical University (The People’s Hospital of Lishui), Zhejiang, 323000 China; 2 Department of Nephrology, Renmin Hospital of Wuhan University, Wuhan, 430000 China; 3 Department of Urology, Shanghai General Hospital, Shanghai Jiao Tong University School of Medicine, No.100 Haining Road, Hongkou District, Shanghai, 200080 China; 4 Department of Urology, The First people’s Hospital of Yancheng (Yancheng First Hospital, Affiliated Hospital of Nanjing University Medical School), No.66 South Renmin Road, Yancheng, 224000 Jiangsu China

**Keywords:** ROC1, NF-κB, Ubiquitination, Metastasis, Bladder cancer

## Abstract

**Background:**

Regulator of cullins 1 (ROC1) is an important catalytic subunit of cullin–RING E3 ligase. Nuclear factor-kappa B (NF-κB) signaling is closely related to tumor invasion and metastasis. Earlier, we reported that ROC1 was associated with a poor prognosis in patients with bladder cancer (BCa). However, it is unclear whether ROC1 is involved in the NF-κB signaling associated with malignant BCa progression.

**Methods:**

The expression of ROC1 and p65 in bladder cancer and paracancerous tissues were detected by immunohistochemistry (IHC). Pearson correlation was used to assess correlation between ROC1 and p65 protein expressions. The wound-healing and transwell assays were used to monitor cell invasion and migration. The effect of ROC1 on the expression of key proteins in the NF-κB signaling was determined by immunofluorescence and western blot (WB). Cycloheximide (CHX), MG132 and immunoprecipitation assays were used to evaluate the effect of ROC1 on the ubiquitination of phosphorylated inhibitor of kappa B alpha (p-IκBα). A lung metastasis mouse model was generated to detect the role of ROC1 in tumor metastasis.

**Results:**

We found that ROC1 was up-regulated in BCa tissues and cell lines, and high ROC1 levels were positively correlated with higher tumour grade, lymph node metastasis, distant metastasis and poor prognosis. Linear-regression analysis showed significant a Pearson correlation between ROC1 and nuclear p65 expression in BCa tissue microarray (TMA) samples. Functional studies demonstrated that ROC1 promoted BCa cell invasion and migration. In vitro and in vivo experiments showed that ROC1 activated NF-κB signaling by enhancing the ubiquitination of p-IκBα, which caused p65 nuclear translocation and promoted the transcription of some metastasis-related target genes, such as urokinase-type plasminogen activator receptor (uPAR), intracellular adhesion molecule 1 (ICAM1), vascular cell adhesion molecule 1 (VCAM1), and matrix metalloproteinase 9 (MMP9), resulting in promoting BCa metastasis.

**Conclusion:**

ROC1 plays an important role in the progression of BCa and serves as a potential diagnostic and therapeutic target for patients with BCa.

**Supplementary Information:**

The online version contains supplementary material available at 10.1186/s13046-021-01935-5.

## Background

Bladder cancer (BCa) has the fourth highest incidence rate of all types of cancers worldwide and the eighth highest mortality rate [1], thus, representing a serious threat to public health. However, relapsed or metastatic BCa lacks treatment. Identifying new metastasis-related gene-therapy targets has become a clinical priority. Abnormal protein metabolism caused by ubiquitin dysregulation is closely related to tumorigenesis and progression [2]. The ubiquitin–proteasome system (UPS) is responsible for the degradation of most proteins [3]. Consequently, ubiquitin modification plays important roles in biological processes such as cell differentiation, apoptosis, DNA-damage repair, immune responses, and stress responses [4]. Protein ubiquitination involves a three-step cascade mediated by ubiquitin-activating (E1), ubiquitin-conjugating (E2), and ubiquitin-ligase (E3) enzymes, where E3 enzyme catalyzes ubiquitin transfer to substrate [5]. However, its exact molecular mechanism has not been fully elucidated. Therefore, elucidating the correlation between abnormal protein ubiquitination and BCa pathogenesis has important clinical significance for developing effective drugs and determining an accurate prognosis for patients with BCa.

The cullin/RING ubiquitin ligase (CRL) family, the largest UPS E3 family, facilitates the degradation of approximately 20% of protein substrates in cells [6]. CRL family is mainly composed of different cullin subunits, such as regulator of cullins (ROC)1 and ROC2, S-phase kinase-associated protein (SKP)1 adaptor protein, and F-box substrate-recognition subunits (SKP2 and beta-transducin repeats-containing protein [β-TrCP]) [7]. CRL family reportedly plays important roles in the occurrence and development of tumors; abnormal ubiquitination of tumor-suppressor protein p53 [8]; cell cycle-related proteins p21 and p27 [9, 10]; transcription factors c-Jun and c-Myc, and hypoxia-inducible factor 1α (HIF-1α) [11–13] can lead to tumorigenesis. The relative specificities of CRL E3 ligases in terms of substrate recognition, makes them potential targets for tumor therapy [14].

ROC1 (ring-box 1 or RBX1) forms the catalytic core of CRL complexes with different cullin subunits [15]. ROC1 is highly evolutionarily conserved and plays a key role in regulating CRL function. It is abnormally high-expressed in the liver, stomach, esophagus, breasts, lungs, and colon malignancies, and is associated with poor clinical prognosis [16–19]. Previously, we found that ROC1 was highly expressed in BCa and its knockdown inhibited BCa cell growth [20]. Further, ROC1 knockdown inhibited CRL activity and triggered p21 and p27 accumulation, leading to G2 phase arrest and cellular senescence [21]. We also found that ROC1 expression was significantly higher in muscular invasive BCa and positively correlated with epithelial–mesenchymal transition (EMT). ROC1 down-regulation caused DEP domain-containing mammalian target of rapamycin (mTOR)-interacting protein (DEPTOR) accumulation, thereby inhibiting mTOR kinase activity. mTOR kinase inhibition can promote the mesenchymal–epithelial transformation and inhibit BCa cell metastasis [22]. These findings provide partial explanation for the important role of ROC1 in BCa progression. However, owing to the diversity of CRL complex-recognized substrates, the understanding of the role of ROC1 in BCa progression is still in its infancy. Notably, some CRLs recognize multiple substrates; β-TrCP [23] not only targets the degradation of β-catenin (which regulates the canonical Wnt-signaling pathway) [24], but also mediates the ubiquitination of phosphorylated inhibitor of kappa B alpha (p-IκBα). Therefore, β-TrCP may play a key role in regulating nuclear factor-kappa B (NF-κB) signaling [25]. Moreover, constitutive NF-κB activation promotes BCa invasion and metastasis, and high nuclear expression of the p65 is associated with poor prognosis [26]. Therefore, in the present study, we explored how ROC1 affects the NF-κB-signaling pathway to promote BCa invasion and metastasis through ubiquitination. The results of this study may provide new evidence for developing future targeted therapies against BCa.

## Methods

### Sample collection and patient follow-up

From January 2007 to October 2014, 46 paraffin-embedded BCa specimens and 10 paired tumor and adjacent normal tissue specimens were obtained from Shanghai General Hospital (Shanghai, China) for TMA construction and IHC analysis. Two pathologists examined and confirmed the tumor and adjacent normal tissues. None of the patients enrolled in the study received other treatments. Tumor staging and grade were evaluated according to the 1973 standards of the World Health Organization and the 2002 TNM system of the American Joint Committee on Cancer. All patients provided written informed consent before participating in the study. This study was approved by the Medical Ethics Committee of Shanghai General Hospital (IRB number: 2013KY004). During the first 5 years of the follow-up period, 56 patients underwent physical and laboratory examinations every 3–6 months, and thereafter every 12 months. All these patients were followed until the study end date (December 30, 2014) or their death. The total survival time was calculated as the period between the dates of diagnosis and death, last known survival date, or study endpoint. The median follow-up time for this study was 35.7 months (range: 3–82 months).

### TMA construction and IHC analysis

TMA construction and IHC were performed as described previously [27]. Briefly, the TMA was incubated with primary anti-ROC1 antibody (1:1000; Abcam, Cambridge, MA, USA) and anti-p65 antibody (1:800; Cell Signaling Technology, Danvers, MA, USA). ROC1-expression levels were analyzed semi-quantitatively using an immunoreactivity-scoring system as described previously [21]. Positive staining was quantified as the integral optical density (IOD)/unit area using ImageJ software (National Institutes of Health, Bethesda, MD, USA), and the mean densities were calculated.

### Cell culture and reagents

Human BCa cell lines (253 J, BIU87, T24, J82, EJ, RT4, and 5637) were purchased from the Chinese Academy of Science (Shanghai, China) and cultured in RPMI-1640 media (Gibco, Gaithersburg, MD, USA) supplemented with 10% fetal bovine serum (FBS; Biological Industries, M.P. Ashrat, Israel) and 1% penicillin–streptomycin. Additionally, NUCs were obtained from fresh bladder tissues of two donors (57 and 64 years old) and cultured in keratinocyte serum-free medium (Gibco) supplemented with 1% FBS and 1% penicillin–streptomycin. LPS and DMSO were purchased from Sigma-Aldrich (St. Louis, MO, USA), and CHX, MG132, and BAY 11–7082 were purchased from MedChemExpress (Monmouth Junction, NJ, USA).

### SiRNA transfection and lentivirus-mediated ROC1 overexpression

Two siRNAs targeting the ROC1 sequence (siROC1–1: GACTTTCCCTGCTGTTACCTAA; siROC1–2: CTGTGCCATCTGCAGGAACCACATT) were synthesized by Genepharm, Inc. (Shanghai, China) and siIκBα was purchased from Thermo Fisher Scientific (Waltham, MA, USA). Cells were transfected with siROC1–1, siROC1–2, and siCONT using the Lipofectamine RNAiMAX transfection reagent (Invitrogen, Carlsbad, CA, USA) to knockdown endogenous ROC1 expression, according to the manufacturer’s instructions. ROC1-Flag-EGFP lentiviral vector and the corresponding empty vector purchased from Shanghai GeneChem Company (Shanghai, China) were used to transduce cells, according to the manufacturer’s instructions. Puromycin (Sigma-Aldrich) was used to select for stably transduced cells, and cells transduced with the empty vector were used as controls. ROC1 expression was verified by qRT-PCR and WB analysis.

### RNA isolation and qRT-PCR analysis

Total RNA was extracted from cells using Spin Column Animal Total RNA Purification Kit (Sangon, Shanghai, China), following the manufacturer’s instructions. Reverse transcription was performed using PrimeScript RT Master Mix (Takara, Shiga, Japan), and qRT-PCR was subsequently performed using PowerUp SYBR Green Master Mix (Thermo Fisher Scientific) and a QuantStudio 7 Flex Real-Time PCR system (Thermo Fisher Scientific). β-actin was used as an internal control. All primers were purchased from Sangon, Inc. The primer sequences are given in Supplementary Table 1.

### Wound-healing and Transwell-invasion assays

Cells were grown in 35-mm culture dishes until they reached 90% confluency, then scratched with a sterile 200-μL pipette-tip. Photographs were taken at 0 and 24 h after scratching. Transwell-invasion assays were performed using 8-μm pore BioCoat Matrigel Invasion chambers (Corning NY, USA), according to the manufacturer’s instructions. Cultured T24 and 5637 cells were trypsinized, resuspended in serum-free RPMI-1640, and added to the upper chambers at a density of 50,000 cells/well. RPMI-1640 with 10% FBS was added to the lower chambers. After a 24-h incubation, the cells were stained with 0.1% crystal violet for 30 min. Unmigrated cells were carefully removed from the upper chamber with a cotton swab and cells that passed through the membrane were counted in four random regions. Three independent experiments were performed.

### Immunofluorescence staining

Cells in the dish were fixed with 4% paraformaldehyde and incubated with a primary antibody against p65 overnight at 4 °C. After washing several times with phosphate-buffered saline (PBS), the cells were incubated with an appropriate fluorophore-conjugated secondary antibody (Abcam) in the dark and counterstained with 4′,6-diamidino-2-phenylindole (DAPI; Beyotime, Shanghai, China). Images were captured using a confocal laser-scanning microscope (Leica TSC SP8, Wetzlar, Germany).

### Protein extraction and WB analysis

Cellular proteins were extracted using RIPA lysis buffer (Thermo Fisher Scientific) and quantified using Pierce BCA Protein Detection Kit (Thermo Fisher Scientific). Lysate proteins were separated by sodium dodecyl sulfate-polyacrylamide gel electrophoresis (SDS-PAGE), transferred to a 0.22-μm or 0.4-μm polyvinylidene fluoride membrane (Millipore, Billerica, MA), and incubated with antibodies against ROC1 (Abcam, ab133565), β-actin (Sangon, D191048), IKKα (Cell Signaling Technology, 11,930), IKKβ (Cell Signaling Technology, 8943), phospho-IKKα/β (Ser176/180, Cell Signaling Technology, 2697), NF-κB/p65 (Cell Signaling Technology, 8242), phospho-NF-κB/p65 (Ser536, Cell Signaling Technology, 3033), IκBα (Cell Signaling Technology, 4814), phospho-IκBα (Ser32, Cell Signaling Technology, 2589), uPAR (Cell Signaling Technology, 12,863), ICAM1 (Cell Signaling Technology, 4915), VCAM1 (Cell Signaling Technology, 39,036), MMP9 (Cell Signaling Technology, 13,667), or ubiquitin (Cell Signaling Technology, 3936). GAPDH (Cell Signaling Technology, 5174), Histone H3 (Cell Signaling Technology, 4499), and β-actin served as loading controls. Binding of the primary antibody was detected by incubating the membranes with a horseradish peroxidase-conjugated secondary antibody, followed by visualization using an enhanced chemiluminescence (ECL) system (Bio-Rad, Hercules, CA, USA).

### Cycloheximide and MG132 assays

CHX or MG132 was added to the culture medium at a final concentration of 30 μM or 20 mM, respectively. CHX group was pretreated with LPS for 2 h. Cell lysates were collected at 0, 2, 4, 8, and 12 h after CHX or MG132 treatment.

### Ubiquitination assays

Pierce Classic Magnetic IP/Co-IP Kit (Thermo Fisher Scientific) was used for p-IκBα immunoprecipitation, according to the manufacturer’s instructions. Then, the supernatants were resolved by SDS-PAGE and subjected to WB analysis with anti-ubiquitin antibody.

### Treatment with NF-κB inhibitor and siIκBα

ROC1-overexpressing cells were treated with BAY 11–7082 (5 μM) or DMSO for 12 h to evaluate their effects on p65 nuclear transport and cell invasion. siIκBα (Thermo Fisher Scientific) was used to transfect ROC1-overexpressing cells to knockdown IκBα, and scrambled siRNA was used as control. Cell invasion and p65 nuclear translocation were detected 48 h after transfection.

### In vivo metastasis assay

Luciferase lentiviral vectors were purchased from GeneChem and transductions were performed according to the manufacturer’s instructions to generate T24 cells double-positive for EGFP and luciferase. After adding d-luciferin to the transduced cells (Yeason, Shanghai, China), the transfection efficiency was verified using a small-animal IVIS instrument (Lumina III, PerkinElmer, Boston, MA, USA). Double-positive T24 cells overexpressing ROC1 (LV-ROC1-OE cells) or transduced with empty vector control (LV-vector cells) were used for in vivo experiments. A single-cell suspension prepared in PBS (200 μL, containing 1 × 10^6^ cells) was injected into the tail veins of 6-week-old male nude mice (nu/nu; Shanghai SLAC Laboratory Animal Co., Ltd., Shanghai, China) (*n* = 10). After 8 weeks, in vivo imaging was performed, the mice were sacrificed, and number of lung-metastatic nodules was counted and subjected to IHC analysis for ROC1, uPAR, ICAM1, VCAM1, and MMP9 expression. All animal experiments were performed with the approval of the Animal Ethics Committee of Shanghai General Hospital. The animal experiments were performed in accordance with relevant guidelines and regulations of the Animal Care and Use Committees at the Shanghai General Hospital.

### Statistical analyses

Results were analyzed using IBM SPSS Statistics software for Windows (version 22.0, SPSS, Inc., Chicago, IL, USA) and expressed as mean ± standard error of the mean of three independent experiments, performed in triplicate. Kaplan–Meier analysis and log-rank tests were used for survival analysis. Correlations between the expression levels of two proteins were analyzed using linear regression. Two-tailed Student’s *t*-test was performed to compare the differences between groups. Multiple comparisons were performed with one-way analysis of variance (ANOVA), followed by Tukey’s post-hoc test. *P* < 0.05 was considered statistically significant.

## Results

### ROC1 positively correlated with nuclear p65 expression in BCa tissues and associated with poor prognosis

**Fig. 1:**
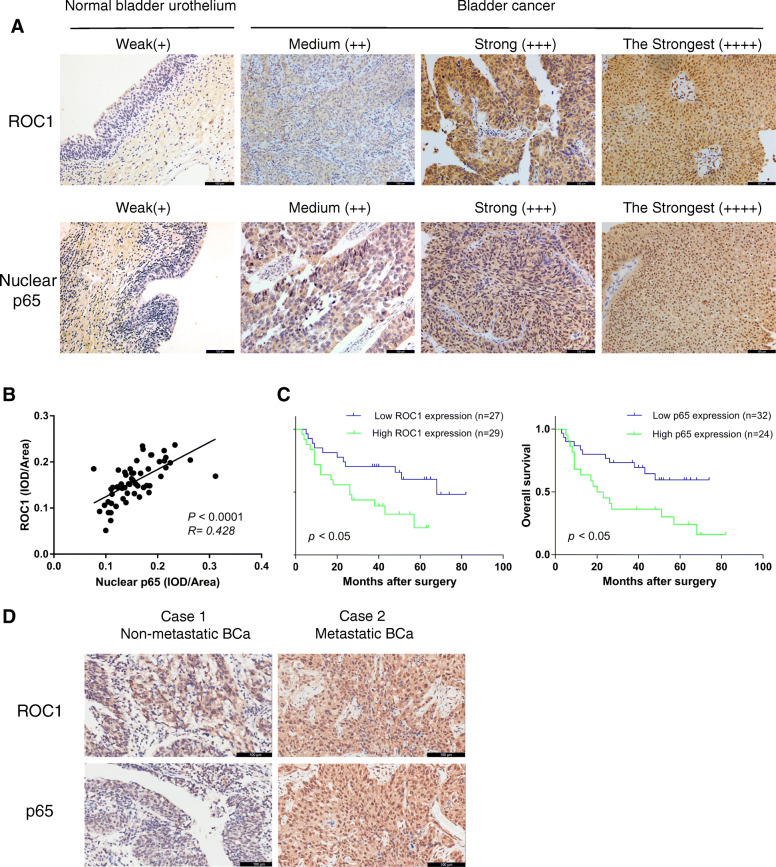
ROC1 expression correlated positively with nuclear p65 expression in BCa tissues and associated with a poor prognosis. **a** Immunostaining with antibodies against ROC1 and p65 (brown) was performed to detect ROC1 and p65 expression in normal bladder urothelial and BCa tissues. TMA sections were counterstained with hematoxylin. Scale bar, 100 μm. **b** Kaplan–Meier analysis for BCa patients with high ROC1 and nuclear p65 expression, and their association with poor overall survival. *P* < 0.05, log-rank test. **c** Linear-regression analysis showed significant a Pearson correlation between ROC1 expression and the mean nuclear p65 IOD/unit area in BCa TMA samples. *P* < 0.0001 R = 0.428. **d** Representative images of ROC1 and p65 IHC staining in BCa metastasis and non-metastasis patients. **e** ROC1 expression in BCa cell lines and NUCs.**P < 0.01 vs Non-metastatic Bca

Here, we performed immunohistochemical (IHC) staining on a TMA, semi-quantitatively detected ROC1 and nuclear p65 expression, and analyzed the correlation between their expression levels (Fig. 1a). ROC1 expression showed a linear positive correlation with nuclear p65 expression (*P* < 0.001, R = 0.428; Fig. 1c). Survival analysis suggested that high expression of each protein was associated with poor prognosis (*P* < 0.05; Fig. 1b). High ROC1 expression was significantly associated with histological grade (*P* = 0.017), lymph node metastasis (*P* < 0.024), and distant metastasis (*P* = 0.020) (Table 1). Furthermore, the expression level of nuclear p65 in patients with BCa metastasis was significantly higher than that in patients without metastasis (Fig. 1d).

**Table 1:** Clinicopathological features of ROC1 expression in bladder cancer

		ROC1 expression		
Parameter	No. of cases	Low	High	*P*-value
Age,years				0.512
< 65	19	8	11	
≥ 65	37	19	18	
Gender				0.566
Male	46	23	18	
Female	10	4	6	
Tumor diameter (cm)				0.089
< 5	42	23	19	
≥ 5	14	4	10	
Histological grade				**0.017**
Low	24	16	8	
High	32	11	21	
Tumor stage				0.063
Ta-1	22	14	8	
T2–4	34	13	21	
LN metastasis				**0.024**
N0	41	24	17	
N1	15	3	12	
Distant metastasis				**0.020**
M0	46	26	20	
M1	10	1	9	

Statistical significance (*P* < 0.05) is shown in bold

### ROC1 expression affected BCa cell migration and invasion

**Fig. 2:**
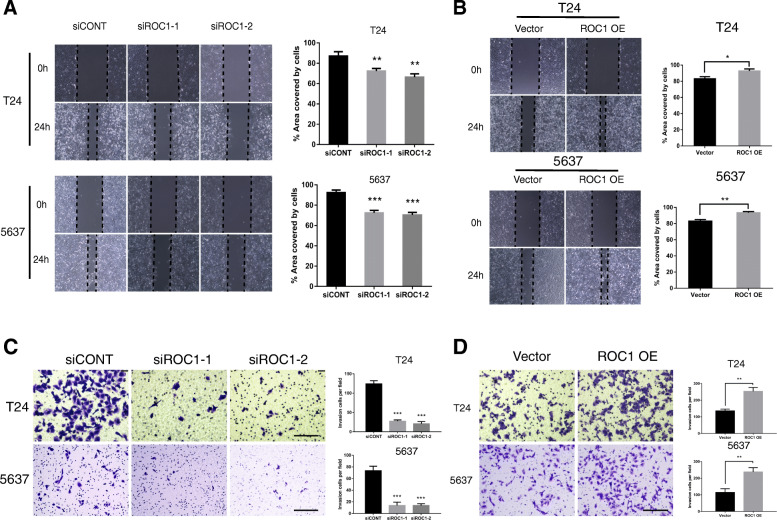
ROC1 promoted the migration and invasion of BCa cell lines. **a**, **b** Wound-healing assays showing that ROC1 overexpression promoted the migration of T24 and 5637 cells, and that ROC1 knockdown showed the opposite effects. The values are expressed as the mean ± standard deviation (SD) of three independent experiments. *P < 0.05, **P < 0.01, ***P < 0.001, two-tailed Student’s *t*-test. **c**, **d** Representative images and summary of Transwell-invasion assay data showing that ROC1 overexpression promoted T24 and 5637 cell invasion, whereas ROC1 knockdown showed the opposite effect. Scale bar, 100 μm. The values are expressed as the mean ± SD of three independent experiments. **P < 0.01, ***P < 0.001, two-tailed Student’s *t*-test

We evaluated ROC1 protein expression in normal urothelium cells (NUCs) and BCa cell lines (253 J, BIU87, T24, J82, EJ, RT4, and 5637) (Fig. 1e) and selected T24 and 5637 cell lines, with highest ROC1 expression, for subsequent experiments. To determine whether ROC1 expression associated with BCa cell invasion and migration, ROC1-overexpression and -knockdown experiments were performed in vitro. Wound-healing (Fig. 2a) and Transwell-invasion assays (Fig. 2c) showed that small-interfering RNA (siRNAs)-mediated ROC1 knockdown significantly inhibited the invasion and migration of T24 and 5637 cells; ROC1 overexpression had the opposite effect (Fig. 2b, d).

### ROC1 promoted NF-κB-signaling activation and target-gene expression

**Fig. 3:**
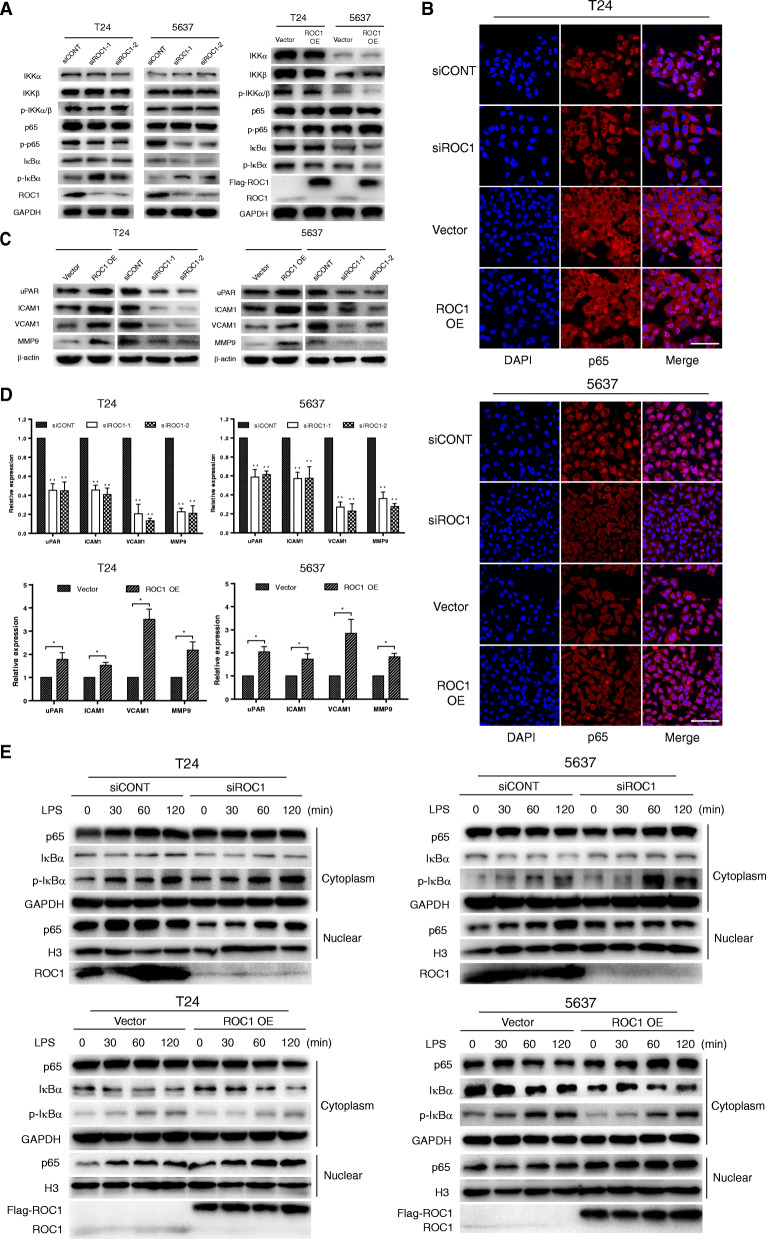
ROC1 overexpression activated NF-κB signaling and promoted the expression of target genes related to metastasis. **a** ROC1 knockdown increased p-IκBα protein expression (left). Conversely, p-IκBα expression decreased following ROC1 overexpression (right). *P < 0.05, **P < 0.01, two-tailed Student’s *t*-test; Tukey’s post-hoc test was performed following one-way ANOVA. **b**, **c** WB and qRT-PCR analysis showed elevated transcript and protein levels of uPAR, ICMA1, VCAM1, and MMP9 in ROC1-overexpressing T24 and 5637 cells. Conversely, ROC1 knockdown showed opposite effects. β-actin served as internal control. The data are represented as the mean ± SD of three independent experiments. *P < 0.05, **P < 0.01, two-tailed Student’s *t*-test; Tukey’s post-hoc test was performed following one-way ANOVA. **d** ROC1 knockdown reduced the nuclear location of p65 (red), whereas ROC1 overexpression increased the nuclear location of p65 (red). Nuclei were counterstained with DAPI (blue). The left picture shows T24 cells and the right picture shows 5637 cells. Cells were counted in six randomly visual fields. Scale bar, 100 μm. The results are expressed as the mean ± SD of three independent experiments. **P < 0.01, ***P < 0.001, two-tailed Student’s *t*-test. **e**. Cells with differing ROC1-expression levels were harvested at different time points after LPS treatment (0, 30, 60, or 120 min), and the nuclear translocation of p65 and other NF-κB-related markers were determined by WB. GAPDH and H3 were used as the cytoplasmic and nuclear markers, respectively

As many target genes of the NF-κB-signaling pathway are closely related to tumor invasion and migration, we explored the effect of ROC1 on NF-κB signaling. First, we detected the expression of NF-κB-signaling markers in T24 and 5637 cells after ROC1 knockdown and overexpression, by WB analysis. After ROC1 RNA interference, p-IκBα levels increased and p-p65 levels decreased, suggesting NF-κB signaling inhibition. ROC1 overexpression showed the opposite effect, suggesting NF-κB signaling activation, although no significant differences were observed in the expression levels of inhibitor of NF-κB kinase subunits (IKKα, IKKβ), p-IKKα/β, p65, or IκBα (Fig. 3a). Subsequently, we detected the expression levels of downstream NF-κB signaling-target genes related to tumor migration and invasion (urokinase-type plasminogen activator receptor [uPAR], intracellular adhesion molecule 1 [ICAM1], vascular cell adhesion molecule 1 [VCAM1], and matrix metalloproteinase 9 [MMP9]) by quantitative real-time polymerase chain reaction (qRT-PCR) and WB (Fig. 3b, c) analysis; their expression levels was significantly higher in ROC1-OE group than that in control group, while, ROC1 knockdown showed the opposite effects.

### Activation of NF-κB signaling by ROC1 was p-IκBα dependent

p-IκBα degradation and p65 nuclear translocation are key factors in NF-κB signal activation. To elucidate the mechanism whereby ROC1 activates NF-κB signaling, we treated BCa cells subjected to ROC1 knockdown or overexpression with NF-κB signaling agonist, lipopolysaccharide (LPS). The number of nuclear p65-positive cells significantly decreased in siROC1 group at 120 min post-LPS treatment, whereas more p65-positive cells were found in ROC1-OE group than control group at 90 min post-LPS treatment (Fig. 3d). We harvested cells after LPS stimulation for 0, 30, 60, or 120 min. Following nucleocytoplasmic separation, we observed a higher cytoplasmic p-IκBα expression in siROC1 group than in control group (siCONT). p-IκBα protein level continued to increase with prolonged LPS stimulation. After 120 min LPS stimulation, siROC1 group showed higher p-IκBα expression, but lower p65 protein expression in the nucleus than control group; ROC1-OE group showed an opposite trend. Additionally, no significant differences were observed in the cytoplasmic expression levels of p65 and IκBα between siROC1 and negative-control siRNA (siCONT) groups, or between empty-vector and ROC1-OE groups (Fig. 3e).

### ROC1 promoted p-IκBα ubiquitination and degradation

**Fig. 4:**
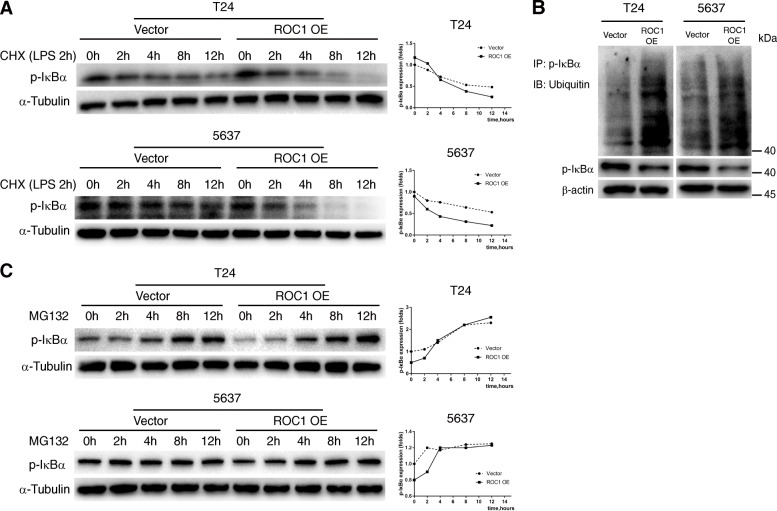
ROC1 promoted p-IκBα ubiquitination and degradation. **a** After a 2 h pretreatment with LPS (1 μg/mL), treatment with CHX (30 μM) showed that ROC1 overexpression accelerated the degradation of p-IκBα. α-Tubulin was used as an internal reference. **b** At 6 h post-treatment with MG132 (10 μM), cell lysates were obtained and immunoprecipitated with an anti-p-IκBα antibody, followed by immunoblotting against ubiquitin. The accumulation of ubiquitinated p-IκBα induced by MG132 was negatively affected by ROC1 expression. β-actin was used as an internal reference. **c**. Treatment with MG132 assays (10 μM) showed that ROC1 overexpression enhanced p-IκBα turnover. α-Tubulin was used as an internal reference

To determine whether ROC1 can promote p-IκBα degradation through the UPS, we first treated ROC1-overexpressing and control cells with protein synthesis inhibitor, cycloheximide (CHX) and proteasome inhibitor, MG132. The p-IκBα half-life in CHX-treated ROC1-OE cells was significantly shorter than that in LPS-pretreated (2 h) control cells (Fig. 4a). In the unstimulated state, about 4 h after MG132 treatment, p-IκBα reached similar levels in both groups (Fig. 4c). To determine whether ROC1 can promote p-IκBα ubiquitination, we treated ROC1-overexpressing and control cells with MG132. After MG132 (10 μM) treatment for 6 h, cell lysates were immunoprecipitated with anti-p-IκBα antibody, followed by WB analysis of ubiquitin. Compared with control group, p-IκBα ubiquitination was higher in ROC1-OE group (Fig. 4b).

### NF-κB inhibition and IκBα knockdown eliminated differences in BCa cell invasiveness induced by ROC1 overexpression

**Fig. 5:**
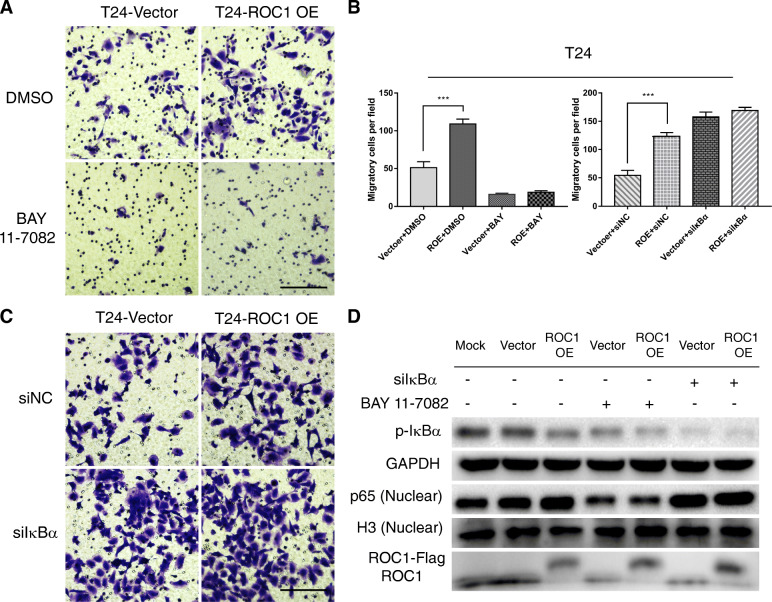
NF-κB inhibition and IκBα knockdown eliminated the difference in invasiveness of BCa cells induced by ROC1 overexpression. **a**, **c** Transwell-invasion assays for ROC1-overexpressing T24 cells treated with BAY 11–7082 (or DMSO) and siIκBα (or siNC, a negative-control siRNA). Representative images of invaded cells are shown, and the results are summarized for each experimental condition. At least three independent experiments were performed, and images of the invaded cells from representative experiments are shown. Scale bar, 100 μm. **b** The histogram shows the number of invaded cells/field after treatment with the indicated chemicals and siRNAs. All results are expressed as the mean ± SD of three independent experiments. ***P < 0.001, two-tailed Student’s *t*-test. **d** WB was performed to detect cytoplasmic p-IκBα and nuclear p65 expression after the cells were treated with the indicated chemicals and siRNAs. H3 and GAPDH were used as nuclear and cytoplasmic markers, respectively

Next, we evaluated the effect of NF-κB inhibition in ROC1-overexpressing BCa (T24) cells. WB revealed that BAY 11–7082 significantly reduced IκBα phosphorylation and inhibited nuclear p65 translocation. Corresponding invasion assays showed that BAY 11–7082 significantly inhibited T24 cell invasion in ROC1-OE and control groups (Fig. 5a, c). Moreover, compared to dimethyl sulfoxide (DMSO)-treated control cells, BAY 11–7082 eliminated the ROC1 overexpression-induced enhanced T24 cell invasiveness. Additionally, siRNA-mediated knockdown of IκBα expression, revealed opposite results in the invasion assays and by WB analysis (Fig. 5b).

### ROC1 overexpression promoted BCa metastasis in vivo

**Fig. 6:**
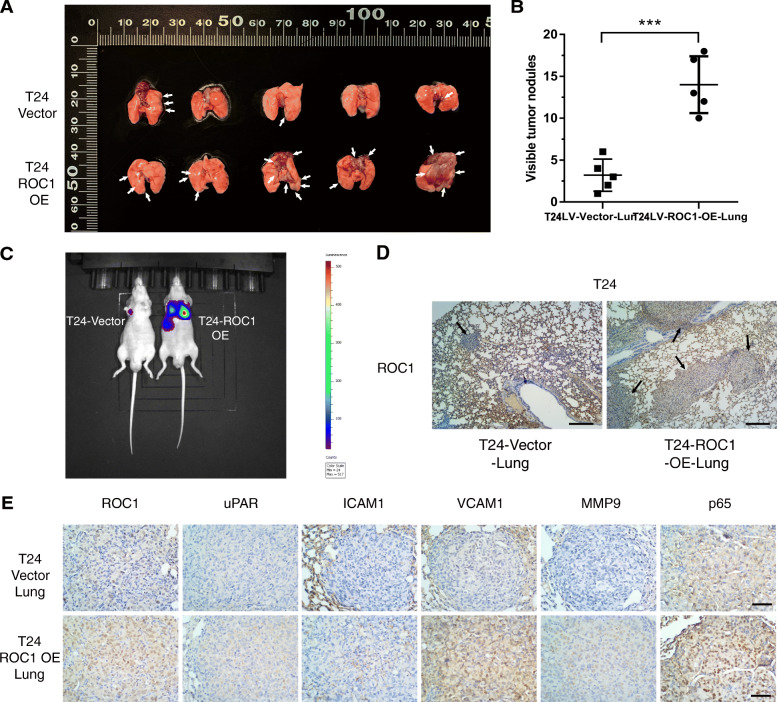
ROC1 overexpression promoted BCa metastasis in vivo. **a**, **b** Representative image and summary of findings pertaining to metastatic nodules in nude mice administered LV-Vector or LV-ROC1-OE T24 cells via tail vein injection. The white arrows indicate pulmonary metastatic nodules. These values are expressed as the mean ± SD of 10 mice. ***P < 0.001, as determined using one-way ANOVA with Tukey’s post-hoc test. **c** Representative bioluminescence images of live animals, taken 8 weeks after tail vein injection. **d** Pathological lung-tissue sections were observed under a light microscope (40×). The black arrows point to lung-metastasis nodules. Scale bar, 1 mm. (E) Representative IHC-staining images of ROC1, uPAR, ICAM1, VCAM1, MMP9, and p65 in lung sections of mice injected with ROC1-overexpressing cells and control cells. Scale bar, 100 μm

To investigate the role of ROC1 in metastasis in vivo, we used a nude mouse model of lung metastasis to verify the effect of ROC1 overexpression. As T24 cells were more tumorigenic than 5637 cells, we selected them for the animal experiments. Enhanced green fluorescence protein (EGFP)- and luciferase-overexpressing T24 cells were transfected with either lentivirus-mediated ROC1 plasmids (LV-ROC1-OE cells) or empty vector control (LV-Vector cells). The cells were injected into the tail veins of nude mice, and lung metastases were observed using an in vivo imaging system. The bioluminescence signals of LV-ROC1-OE group were significantly higher than those of LV-Vector group (Fig. 6c). After 8 weeks, the mice were sacrificed and their lung tissues were analyzed. The pulmonary nodules in the stained lung sections of mice in both groups were observed (Fig. 6a). Compared with LV-Vector group (3.2 ± 1.92), LV-ROC1-OE group (14 ± 3.39; *P* < 0.001) had more lung metastatic nodules (Fig. 6b, d). The expression levels of uPAR, ICAM1, VCAM1, MMP9, and p65 in lung-nodule tissues, determined by IHC, were elevated in LV-ROC1-OE group (Fig. 6e). These results were consistent with the in vitro data, suggesting that ROC1 overexpression may promote tumor metastasis mediated by NF-κB-signaling activation.

## Discussion

This study found that ROC1 played an important role in the malignant progression of BCa. First, we confirmed positive correlation between ROC1 and nuclear p65 expression, suggesting that ROC1 may be related to the constitutive activation of the NF-κB pathway. Moreover, aberrantly high ROC1 expression was associated with aggressive BCa tumor features and poor prognosis. ROC1 also promoted BCa cell migration and invasion. We found that ROC1 overexpression promoted the transcription of NF-κB-pathway target genes, uPAR, VCAM1, ICAM1, and MMP9, which enhanced BCa cell migration and invasion. The results of MG132 and CHX treatment indicated that the degradation of p-IκBα was mainly regulated by UPS, and ROC1 overexpression enhanced the turnover of p-IκBα. Furthermore, we discovered that ROC1 regulated the NF-κB-signaling pathway by controlling p-IκBα ubiquitination. Finally, we verified the role of ROC1 and NF-κB signaling in BCa progression in vivo.

Tumor metastasis is a complex process that involves the interactions of various proteins and multiple signaling pathways. In addition to regulating BCa cell EMT via DEPTOR–mTOR axis, ROC1 may also cooperate with NF-κB signaling in BCa metastasis. The nuclear p65 is believed to be constitutively expressed in human prostate [28], breast [29], liver [30], colon cancers [31] and mediates tumor metastasis. By analyzing 116 BCa patients’ tissues, Levidou et al. [26] found that BCa malignancy was closely related to nuclear p65 expression, suggesting that its constitutive expression is related to BCa progression. Similarly, we found that patients with higher p65 nuclear expression had a worse prognosis. Our research further confirmed the important role of NF-κB signaling in BCa progression.

NF-κB signaling is involved in numerous biological processes such as immune and inflammatory responses, proliferation, apoptosis, and EMT [32]. In the canonical NF-κB pathway, p65 is mostly present in the cytoplasm as an inactive complex that binds to IκBα. On receiving relevant signals, IKK phosphorylates IκBα, which is then ubiquitinated and subsequently degraded by the 26S proteasome. Finally, p65 rapidly enters the nucleus and activates gene transcription [33]. Therefore, regulation of the IκBα–p65 interaction is a key rate-limiting step in controlling NF-κB-signaling activity. NF-κB-signaling activation can promote tumor progression by chronically stimulating cancer cell proliferation, inhibiting cell death, and promoting the accumulation of mutations; it can up-regulate the expression of transcription factors, such as TWIST1 and SNAIL, to promote EMT [34] and induce the expression of uPAR, cell-adhesion molecules, and MMPs, which help cancer cells escape from the circulation [35]. NF-κB-signaling activation can also stimulate HIF-1α expression, thereby enhancing the hypoxic adaptation of tumor cells and the early survival of metastasis-initiating cells [36]. Our findings suggested that ROC1 overexpression accelerates ubiquitination-dependent degradation of p-IκBα in BCa cells, promotes nuclear translocation of p65, and activates expression of target genes involved in tumor metastasis. Furthermore, our observation that BAY 11–7082, a small-molecule NF-κB signaling inhibitor, effectively eliminated ROC1 overexpression-induced BCa cell invasion, suggests that NF-κB-signaling inhibition may be an effective treatment strategy for BCa with high ROC1 expression.

Notably, β-TrCP activates NF-κB signaling through ubiquitinated p-IκBα to promote tumor metastasis, as seen in oral squamous cell carcinoma [37]. Combined with our current results, these data suggest that ROC1 may be an important component in β-TrCP complex. However, it is important to realize that ROC1 is a common component of many CRL E3 ligases and can combine with other CRL substrate-recognition subunits [38]. Consequently, one E3 ligase type could transfer ubiquitin to different substrates, and the same substrate could be recognized by several E3 ligases [39]. Therefore, the regulatory mechanism whereby ROC1 promotes BCa progression is complex. The interactions between ROC1 and other potential targets, and new modes of post-translational modifications to ROC1 itself should be further explored. There are some limitations of the present study. First, T24 and 5637 cells showed higher expression of ROC1 and were used for performing ROC1 knockdown experiments and further cellular experiments. We did not perform the ROC1 overexpression experiment using other cell line. Second, only T24 cells were carried out for NF-κB inhibition and IkBa knockdown studies, and 5637 cells should be involved. These limitations will be a part of our future research.

## Conclusions

We found that ROC1 played an important role in BCa progression by controlling p-IκBα ubiquitination and regulating NF-κB signaling. The results may provide new insights regarding the relationship between ROC1 and NF-κB signaling that should help in understanding the tumorigenic mechanism of BCa and aid in developing targeted therapies.

## Supplementary Information

**Additional file 1.**

## Data Availability

Please contact author for data requests.

## Abbreviations

ROC1: Regulator of cullins 1
BCa: Bladder cancer
NF-κB: Nuclear factor-kappa B
p-IκBα: Phosphorylated inhibitor of kappa B alpha
uPAR: Urokinase-type plasminogen activator receptor
ICAM1: Intracellular adhesion molecule 1
VCAM1: Vascular cell adhesion molecule 1
MMP9: Matrix metalloproteinase 9
UPS: Ubiquitin proteasome system
CRL: Cullin/RING ubiquitin ligase
ROC: Regulator of cullins
SKP: S-phase kinase-associated protein
β-TrCP: Beta-transducin repeats-containing protein
HIF-1α: Hypoxia-inducible factor 1α
EMT: Epithelial–mesenchymal transition
IHC: Immunohistochemical
TMA: Tissue microarray
NUCs: Normal urothelium cells
LPS: Lipopolysaccharide
CHX: Cycloheximide
DMSO: Dimethyl sulfoxide
qRT-PCR: Quantitative real-time polymerase chain reaction
RPMI: Roswell park memorial institute
SDS-PAGE: Sodium dodecyl sulfate-polyacrylamide gel electrophoresis
